# Salidroside protects against foam cell formation and apoptosis, possibly via the MAPK and AKT signaling pathways

**DOI:** 10.1186/s12944-017-0582-7

**Published:** 2017-10-10

**Authors:** Jing Ni, Yuanmin Li, Weiming Li, Rong Guo

**Affiliations:** 1 Department of Cardiology, Shanghai Tenth People’s Hospital, Tongji University School of Medicine, 301 Yan Chang Zhong Road, Shanghai, 200072 China; 2 Department of Cardio-Thoracic Surgery, Shanghai Tenth People’s Hospital, Tongji University School of Medicine, 301 Yan Chang Zhong Road, Shanghai, 200072 China

**Keywords:** THP1, Salidroside, Foam cell, Akt pathway, MAPK pathway

## Abstract

**Background:**

Foam cell formation and apoptosis are closely associated with atherosclerosis pathogenesis. We determined the effect of salidroside on oxidized low-density lipoprotein (ox-LDL)-induced foam cell formation and apoptosis in THP1 human acute monocytic leukemia cells and investigated the associated molecular mechanisms.

**Methods:**

THP1-derived macrophages were incubated with salidroside for 5 h and then exposed to ox-LDL for 24 h to induce foam cell formation. Cytotoxicity, lipid deposition, apoptosis, and the expression of various proteins were tested using the CCK8 kit, Oil Red O staining, flow cytometry, and western blotting, respectively.

**Results:**

Ox-LDL treatment alone promoted macrophage-derived foam cell formation, while salidroside treatment alone inhibited it (*p* < 0.05). The number of early/late apoptotic cells decreased with salidroside treatment in a dose-dependent manner (p < 0.05). Salidroside dramatically upregulated nuclear factor erythroid 2-related factor 2, but had no effect on heme oxygenase-1 expression; moreover, it markedly downregulated ox-LDL receptor 1 and upregulated ATP-binding cassette transporter A1. Salidroside also obviously decreased the phosphorylation of JNK, ERK, p38 MAPK, and increased that of Akt. However, the total expression of these proteins was not affected.

**Conclusion:**

Based on our findings, we speculate that salidroside can suppress ox-LDL-induced THP1-derived foam cell formation and apoptosis, partly by regulating the MAPK and Akt signaling pathways.

## Background

Atherosclerosis is a major pathological process for coronary artery disease (CAD), because of which it has attracted significant research interest during recent year [1]. In the early stages of atherosclerosis, oxidization of LDL results in the recruitment of monocyte-derived macrophages to the blood vessel [2]; these macrophages then devour excessive cholesterol and form foam cells [3]. Foam cell formation and apoptosis can contribute to atherosclerotic plaque rupture and accelerate the development of atherosclerosis [4]. Therefore, mitigation of ox-LDL-induced pathological responses has been believed to be an effective approach for atherosclerosis treatment.

Nuclear factor erythroid 2-related factor 2 (Nrf2), a major anti-oxidative transcription factor, regulates the redox balance and metabolism in cells [5, 6]. Under oxidative stress, mitogen-activated protein kinase (MAPK), phosphatidylinositide 3-kinases (PI3ks), as well as reactive oxygen species (ROS) can phosphorylate Nrf2 and promote the dissociation of Nrf2 and Keap1 [7, 8]. Nrf2 can then translocate to the nucleus, upregulate the antioxidant response element (ARE) sequence of phase II detoxification, and translate downstream detoxification enzymes such as heme oxygenase (HO1) [9, 10]. HO1 is a rate-limiting enzyme, which mediates heme catabolism. Its metabolites such as Fe^2+^, carbon monoxide (CO), and biliverdin are involved in inflammatory processes and oxidative tissue damage [10]. Ding et al. [11] reported that dietary Nrf2 activators could alleviate the atherogenic process.

Many proteins such as ATP-binding cassette transporter A1 (ABCA1) and lectin-like oxidized low-density lipoprotein receptor-1 (LOX1) are involved in the regulation of lipid homeostasis [12]. ABCA1 is a member of the transporter family and mediates cholesterol efflux from macrophages to lipid-free apoA-I [13, 14]. It also suppresses inflammatory responses and affects atherosclerosis through different metabolic pathways [15]. LOX1, an important ox-LDL receptor, is associated with ox-LDL-induced cytotoxicity [16, 17]. Some studies have shown that LOX1 is one of the earliest markers of the atherosclerotic process [18]. These data led us to believe that alterations in the expression of ABCA1 and LOX1 may inhibit THP1-derived foam cell formation.

Now-a-days, synthetic and natural antioxidants are widely used to treat oxidative-related diseases including diabetes mellitus, pulmonary fibrosis, hypertension, and atherosclerosis [19, 20], but synthetic antioxidants have been found to have deleterious side effects [21–23]. Therefore, finding natural antioxidants that can induce the expression of antioxidant genes has garnered more attention [24]. *Rhodiola rosea*, a member of the Crassulaceae family, grows in plateau regions and is widely recognized in the Chinese traditional medicine system. Salidroside, a major active ingredient extracted from *Rhodiola rosea*, has been found to exert anti-oxidative stress [25], anti-inflammation [26], and anti-ischemia-reperfusion injury effects [27]. Salidroside treatment has been found to appreciably attenuate atherosclerotic plaque formation and to decrease the expression of MCP1, ICAM-1, and VCAM-1 in the aortic tissue in an LDL−/− mice model [28]. However, the molecular mechanisms underlying this effect of salidroside have not been not well understood so far. Therefore, in the present study, we aimed to determine the effect of salidroside on ox-LDL-induced THP1-derived foam cell formation and apoptosis. Furthermore, we investigated whether the MAPK and Akt pathways are involved in this effect. The fruitful work presented here provides a potential strategy for atherosclerosis therapy.

## Materials and methods

### Materials

Salidroside (≥98% purity) was purchased from Melonepharma (Dalian, China). RPMI 1640, fetal bovine serum (FBS), and antibiotics (streptomycin/ penicillin) were purchased from BRL Life Technologies (Grand Island, NY). The THP1 human leukemia cell line was obtained from the Institute of Biochemistry and Cell Biology (Shanghai Institute for Biological Science, the Chinese Academy of Sciences, Shanghai, China). Phorbol 12-myristate 13-acetate (PMA) and ox-LDL were bought from Sigma-Aldrich (USA). The Oil Red O staining kit was bought from Beijing Noble Rider Technology Co., Ltd. (Beijing, China). The kits for flow cytometry, the CCK8 assay, and the BCA protein assay were purchased from Beyotime Institute of Biotechnology (Haimen, China). Salidroside and ox-LDL were diluted with DMSO and PBS respectively.

### Cell culture

THP1 cells were cultured in RPMI 1640 medium supplemented with 1% penicillin and streptomycin and 10% FBS in a humidified atmosphere of 5% CO2 at 37 °C. The cells were then differentiated into macrophages by adding PMA (2.5 μL) for 24 h. After differentiation, the cells were pre-stimulated with salidroside (0.1, 1, 10 μM) for 5 h and then stimulated with ox-LDL (150 μg/mL) for 24 h at 37 °C in 5% CO2.

### Cell viability

The THP1 cells (105 cells/mL) were plated onto 96-well plates and treated with PMA (2.5 μL) for 24 h at 37 °C in a 5% CO2 incubator. Then, cells were incubated with salidroside for 5 h and stimulated with ox-LDL. Twenty-four hours later, the supernatants were collected and treated with the CCK8 reagent for 2 h. Cell viability was then measured using a microplate reader at 450 nm (Bio-Tek, Winooski, VT, USA).

### Oil red O staining

The THP1 macrophages were pre-incubated with salidroside (0.1, 1, 10 μM) for 5 h and then treated with ox-LDL (150 μg/mL) for 24 h. The cells were then fixed in 4% paraformaldehyde for 20 min at room temperature and washed with PBS. Next, the cells were stained with 0.5% Oil Red O solution for 30 min. Finally, the cells were washed with PBS to remove the unbound dye, and sections were photographed using a fluorescence microscope (Leica DMI6000, Leica, Germany).

### Analysis of apoptosis by flow cytometry

The THP1 cells were seeded into 6-well plates for 24 h and then exposed to salidroside (0.1, 1, 10 μM) for 5 h, followed by treatment with ox-LDL (150 μg/mL) for 24 h. Subsequently, the cells were washed thrice in ice cold PBS. After the addition of binding buffer (195 μL), FITC-labeled Annexin V (5 μL), and propidium iodide (10 μL), the samples were incubated at room temperature for 15 min on ice in the dark. The rate of apoptosis was then determined using a flow cytometer (EPICS-XL, Beckman Coulter, Fullerton, USA).

### Measurement of biomarkers of oxidative stress

Cells were pretreated with or without 0.1–10 μM salidroside for 24 h followed by stimulation with ox-LDL (150 μg/mL) for 30 min. The THP1 cells were collected and incubated on ice in 500 μL of cell lysis buffer (1 mM EDTA, 10 mg/mL aprotinin,

0.5 mg/mL leupeptin, 0.7 mg/mL pepstatin, and 0.5 mM PMSF, pH 7.0). After centrifugation at 10000×g for 5 min, supernatant was collected to determine malonyldialdehyde (MDA), superoxide dismutase (SOD) and glutathione (GSH) by using commercial kits (Jian Cheng Biological Engineering Institute, Nanjing, China).

### Western blotting analysis

Total protein was extracted and the levels were determined using a BCA Protein Assay Kit. Equal amounts of total protein (50 μg) were loaded and then separated by sodium dodecyl sulfate polyacrylamide gel electrophoresis (SDS-PAGE) and transferred to PVDF membranes. The membranes were then blocked in 5% BSA for 1.5 h, followed by overnight incubation at 4 °C with the following primary antibodies: anti-β-actin (1: 1000 dilution), anti-ABCA1 (1: 1000 dilution), anti-Nrf2 (1: 1000 dilution), anti-HO1 (1: 1000 dilution), and anti-LOX1 (1: 1000 dilution) (purchased from Santa Cruz Inc., California, USA). Anti-p38 MAPK (1: 1000 dilution), anti-p-p38 MAPK (1: 1000 dilution), anti-ERK1/2 (1: 1000 dilution), anti-p-ERK1/2 (1: 1000 dilution), anti-JNK (1: 1000 dilution), anti-p-JNK (1: 1000 dilution), anti-Akt (1: 1000 dilution), and anti-p-Akt (1: 1000 dilution) antibodies were purchased from Cell Signaling Technology (Danvers, MA, USA). The secondary antibodies, horseradish peroxidase (HRP)-labeled goat-anti-rabbit and goat-anti-mouse IgG, were purchased from Wuhan Boster Bio-engineering Co. Ltd. (Wuhan, China). Blots were processed for enhanced chemifluorescence using a Pierce ECL western blotting substrate (Thermo Scientific Pierce, Rockford, IL, USA).

### Statistical analysis

All the experiments were repeated at least three times, and the data are expressed as the mean ± standard deviation. All statistical analyses were performed using Graphpad Prism 5.0 software (GraphPad Software, San Diego, CA, USA). Inter-group differences were ana¬lyzed by one-way analysis of variance (ANOVA), followed by post-hoc tests. *p* < 0.05 was considered to indicate a statis-tically significant difference.

## Results

### Salidroside treatment prevents ox-LDL-induced toxicity in THP1 cells

**Fig. 1:**
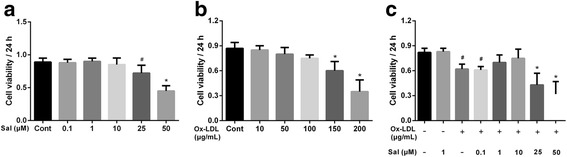
Salidroside prevents ox-LDL-induced decrease in the viability of THP1 cells. **a** THP1 cells were exposed to salidroside at different concentrations (0.1, 1, 10, 25, 50 μM) for 24 h, and cell viability was measured using the CCK8 assay; (**b**) THP1 cells were exposed to ox-LDL at different concentrations (10, 50, 100, 150, 200 μg/mL) for 24 h, and cell viability was measured using the CCK8 assay; (**c**) THP1 cells were incubated with salidroside (0.1, 1, 10, 25, 50 μM) for 5 h, followed by treatment with 150 μg/mL ox-LDL for another 24 h. Data are expressed as mean ± standard deviation (SD). # *p* < 0.05 indicates significant difference from the control group. * *p* < 0.01 indicates significant difference from the control group

To determine the cytotoxic effect of salidroside on THP1 cells, cell viability was tested using the CCK8 assay. As shown in Fig. 1, the viability was significantly decreased when the cells were treated with salidroside at concentrations of 25 or 50 μM (*p* < 0.05, Fig. 1a). Therefore, we did not use salidroside at these higher concentrations for our subsequent experiments. The THP1 cells were then exposed to ox-LDL at various concentrations (0 to 200 μg/mL) for 24 h. While treatment with ox-LDL at 150 μg/mL and 200 μg/mL markedly decreased the cell viability (when cells were not pre-treated with salidroside) (Fig. 1b), treatment with salidroside and ox-LDL induced a remarkable increase in the viability of THP1 cells (Fig. 1c). This result confirmed that treatment with salidroside prevented the ox-LDL–induced decrease in THP1 cell viability.

### Salidroside alleviates ox-LDL–induced foam cell formation in THP1 cells

**Fig. 2:**
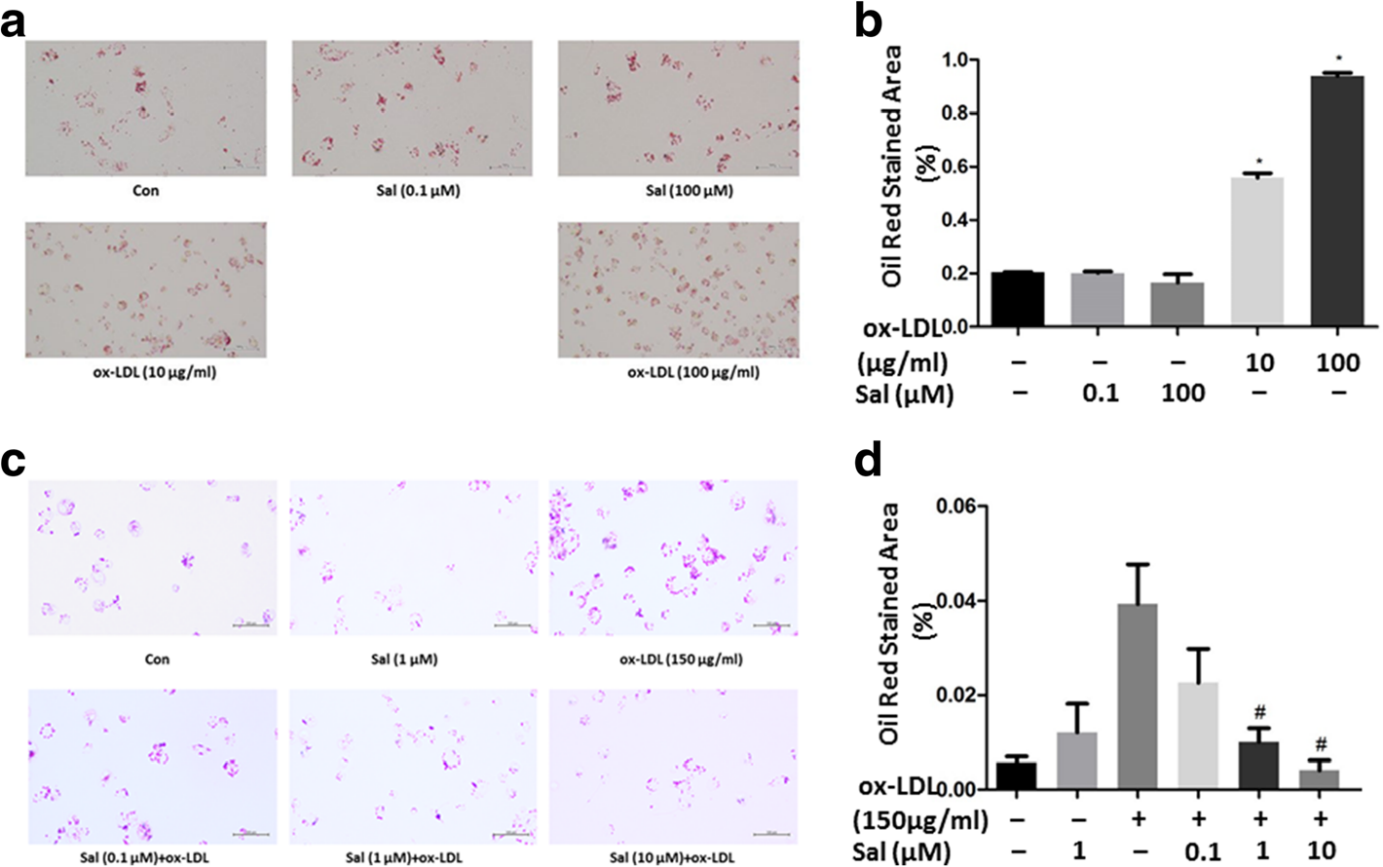
Foam cell formation after 24 h of salidroside pre-treatment. (**a**) and (**b**) Oil red staining areas were showed no differences between salidroside-tearted cell groups. However, ox-LDL significantly increased foam cell formation at 10 and 100 μg/mL; (**c**) THP1 cells were incubated with salidroside (0.1, 1, 10 μM) followed by treatment with 150 μg/mL ox-LDL; (**d**) Quantitative analysis of foam cell formation. Data are expressed as mean ± standard deviation (SD). Red: Oil red O-positive cells; Scale bars indicate 500 μm; # *p* < 0.05 indicates significant difference from the control group. * *p* < 0.01 indicates significant difference from the control group

**Fig. 3:**
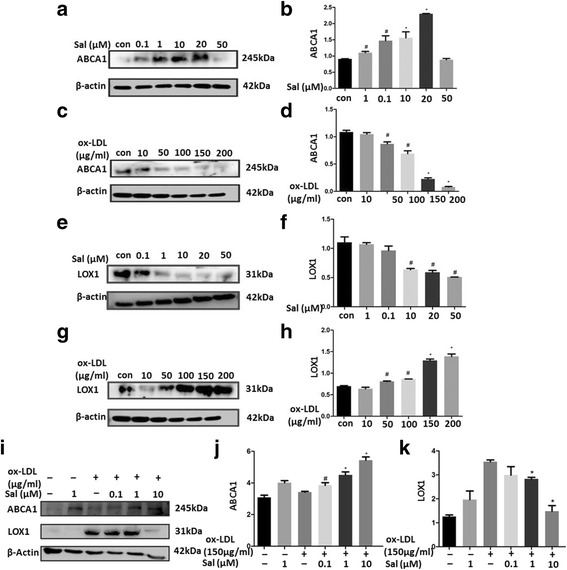
Salidroside pretreatment affects ABCA1 and LOX1 levels in ox-LDL–induced THP1 cells. (**a**) Protein expression of ABCA1 in salidroside-treated THP1 cells was analysed by western blotting. β-Actin was used as the control; (**b**) Quantification of ABCA1 protein expression in salidroside-treated THP1 cells; (**c**) Protein expression of ABCA1 in ox-LDL-treated THP1 cells was analysed by western blotting. β-Actin was used as the control; (**d**) Quantification of ABCA1 protein expression in ox-LDL–stimulated THP1 cells; (**e**) Salidroside treatment significantly decreased LOX1 expression in a dose-dependent manner; (**f**) Quantification of LOX1 protein expression in salidroside–incubated THP1 cells; (**g**) Ox-LDL incubation significantly increased LOX1 expression in a dose-dependent manner; (**h**) Quantification of LOX1 protein expression in the ox-LDL–stimulated THP1 cells; (**i**) to (**k**) ABCA1 protein expressions were restored and LOX1 protein expressions were reduced with salidroside treatment in a dose-dependent manner. Data are presented as mean ± standard deviation (SD). # *p* < 0.05 indicates significant difference from the control group. * *p* < 0.01 indicates significant difference from the control group

To verify the effect of salidroside on lipid homeostasis in THP1 cells, we evaluated the foam cell formation by Oil Red O staining and found that ox-LDL treatment alone significantly increased foam cell formation (when the cells were not pre-treated with salidroside), while pre-treatment with salidroside (*p* < 0.05) significantly reduced foam cell formation in a dose-dependent manner (Fig. 2). We also tested the protein expression of ABCA1 and LOX1, which are known to regulate lipid metabolism. Western blotting revealed that salidroside pre-treatment significantly increased ABCA1 levels (Fig. 3a to d, i and j, *p *<0.05) and decreased LOX1 levels (Fig. 3e to h, *p*<0.05) in the ox-LDL–treated THP1 cells. This finding showed that salidroside treatment could inhibit the ox-LDL–induced foam cell formation in THP1 cells (Fig. 3i to k, *p* < 0.05).

### Effect of salidroside on intracellular Nrf2 and HO1 levels

**Fig. 4:**
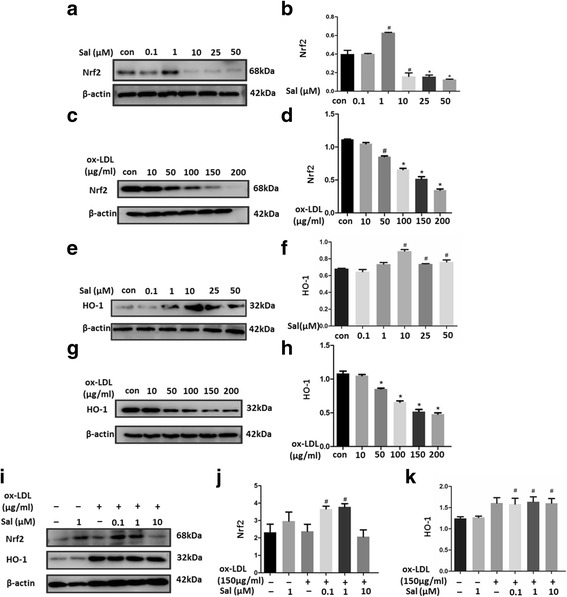
Effect of salidroside pre-treatment on Nrf2/HO1 pathway. (**a**) to (**d**) Protein expressions of Nrf2 and HO1 in THP-1 cells treated with salidroside were analyzed by western blotting. β-Actin was used as the control; (**e**–**h**) Protein expressions of Nrf2 and HO1 in THP-1 cells analyzed by western blotting. β-Actin was used as the control; (**i**–**k**) Nrf2 and treated with ox-LDL were HO1 levels were increased with salidroside treatment in a dose-dependent manner. Data are presented as mean ± standard deviation (SD). # *p* < 0.05 indicates significant difference from the control group. * *p* < 0.01 indicates significant difference from the control group

To investigate whether salidroside mediates the expression of anti-oxidative enzymes, the expression of Nrf2 and HO1 was determined by western blotting. Our results showed that treatment with ox-LDL alone (when cells were not pre-treated with salidroside) significantly decreased Nrf2 expression; however, treatment with salidroside (only at concentration of 0.1 and 1 μM) increased the expression of Nrf2 (*p* < 0.05, Fig. 4). In contrast, pre-treatment with salidroside at 10 to 50 μM decreased Nrf2 expression (Fig. 4a and b). Treatment with salidroside (0.1, 1, 10 μM) alters Nrf2 and HO1 expression in the THP1 cells treated with ox-LDL (Fig. 4i to k). These results suggested that salidroside-mediated alleviation of oxidative stress might be related to the upregulation of Nrf2/HO1 pathway.

### Changes in oxidative stress biomarkers

**Fig. 5:**
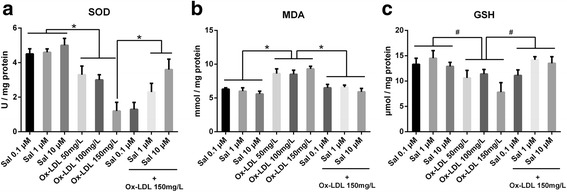
Effect of salidroside on redox parameters after ox-LDL stimulation. (**a**) and (**c**) The levels of SOD and GSH were significantly lower in the cells  incubated with ox-LDL than in salidroside-treated cells; (**b**) The levels of MDA were significantly higher in THP1 cells incubated with ox-LDL compared with cells treated with salidroside. # *p* < 0.05 indicates significant difference between two groups. * *p* < 0.01 indicates significant difference between two groups

The levels of oxidative stress biomarkers of THP1 cells were significantly different (all *p* < 0.05). Figure 5 shows the comparison of SOD, MDA and GSH levels in the supernatant of both salidroside-treated and non-treated THP1 cells. The levels of SOD and GSH were significantly lower in the cells incubated with ox-LDL than in salidroside-treated cells, whereas the levels of MDA were significantly higher in THP1 cells incubated with ox-LDL compared with cells treated with salidroside (*p* < 0.05).

### Salidroside inhibits ox-LDL–induced apoptosis in THP1 cells

**Fig. 6:**
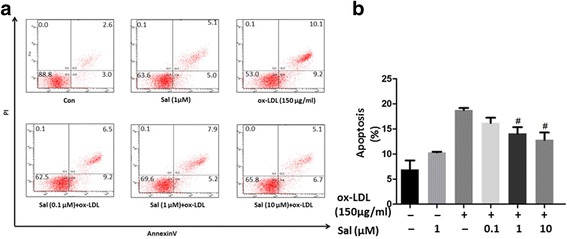
Effect of salidroside pretreatment on ox-LDL–induced THP1 cell apoptosis. (**a**) THP1 cell apoptosis was determined by flow cytometry. The X and Y axes represent Annexin V and PI fluorescence, respectively; (**b**) The mean percentage of apoptotic THP1 cells measured by flow cytometry. THP1 cells were treated with or without salidroside for 5 h, followed by treatment with ox-LDL (150 μg/mL) for another 24 h. Data are expressed as mean ± standard deviation (SD). # *p* < 0.05 indicates significant difference from the control group

To study whether salidroside has an effect on ox-LDL–induced cell apoptosis, Annexin V/propidium iodide double-labeling was performed. As shown in Fig. 6, the proportions of cells in early and late apoptosis were markedly higher in the group treated only with ox-LDL. In contrast, pre-treatment with salidroside significantly reduced the ox-LDL–induced apoptosis in a dose-dependent manner (*p* < 0.05), which confirmed that salidroside could protect against ox-LDL–induced apoptosis in THP1 cells.

### Salidroside has influences on MAPK and Akt phosphorylation

**Fig. 7:**
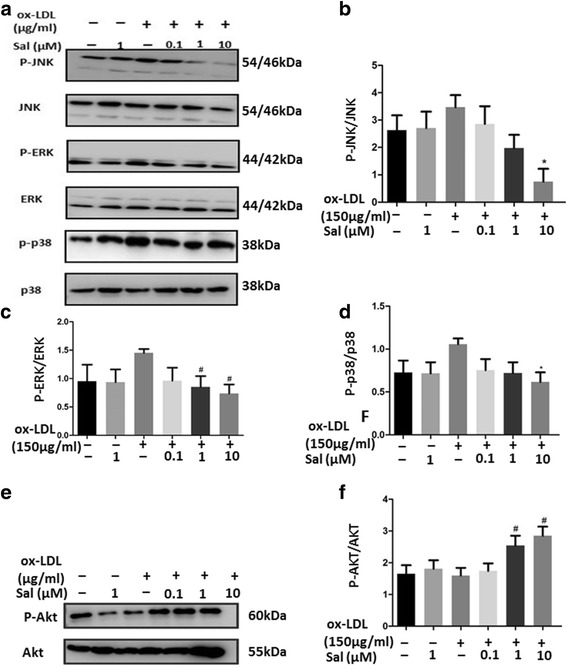
Salidroside regulates MAPK and Akt phosphorylation in THP1 cells. **a** Western blot analysis of p-JNK, total JNK, p-ERK, total ERK, p-p 38, and total p38 levels. β-Actin was used as the control; (**b–d**) Quantification of p-JNK, total JNK, p-ERK, total ERK, total p38, and p-p38 expression; (**e**) Western blot analysis of p-Akt and total Akt levels; (**f**) Quantification of p-Akt, and total Akt expression. Data are presented as mean ± standard deviation (SD). # *p* < 0.05 indicates significant difference from the control group. * *p* < 0.01 indicates significant difference from the control group

In THP-1 cells, the protein expressions of phosphor-JNK 1/2, phospho-ERK 1/2 and phospho-p38 MAPK increased after incubation with ox-LDL for 24 h (Fig. 7a). The expressionsof total JNK, ERK and p38 MAPK were not affected by salidroside treatment, and the expressions of phosphor-JNK, phospho-ERK and phospho-p38 MAPK were reduced following treatment with salidroside pre-treatment for 24 h (Fig. 7a to d).

To investigate the effect of salidroside on the PI3K/Akt signalling pathway, protein expression of phospho-Akt was measured by immunoblotting (Fig. 7). The protein expression of phospho-Akt was markedly increased by incubation of cells with 150 μg/mL ox-LDL for 24 h. This effect was significantly attenuated by pre-treatment with salidroside at 1 and 10 μM (*p* < 0.05, Fig. 7e and f).

## Discussion

During the recent years, many investigators have paid attention to phytochemicals, which are regarded as important sources for drug development [29, 30]. Salidroside, a phenol glycoside, is the main ingredient of *Rhodiola rosea* L [31]. In this study, we found that ox-LDL, a frequently used oxidative stress agent, led to loss of viability in THP1 cells. However, pretreatment with salidroside alleviated the ox-LDL–induced decrease in the viability of THP1 cells, which are an excellent macrophage model system [32]. Our results indicate that salidroside inhibits foam cell formation and apoptosis by activating the Akt and MAPK pathways in THP1 cells (Fig. 7). Based on our findings, we believe that salidroside might prove to be a potential bioactive agent for the treatment of atherosclerosis in the future.

It is well known that ox-LDL can induce oxidative stress, which may lead to the occurrence and development of atherosclerosis [33]. Apoptosis is a regulated type of cell death, in which a cell effectively executes its own demise in a programmed manner. Some reports have indicated that oxidative stress plays a critical role in apoptosis [34, 35]. Seimon et al. [36] confirmed that foam cell apoptosis could promote the development of the necrotic core, which plays an important role in plaque disruption in coronary arteries. Therefore, we found it worthy to further evaluate the effect of salidroside on ox-LDL–induced oxidative damage in THP1 cells. The formation of macrophage-derived foam cells represents the initiation of atherosclerosis [37, 38]. Under pathological conditions, cholesterol efflux appears to be the major factor in the maintenance of cholesterol homeostasis in macrophages [39]. ABCA1 is known as a key transporter for cholesterol efflux, which releases free cholesterol from macrophages to apoA1 [40]. LOX1 is associated with plaque instability. Thakkar et al. [41] reported that ox-LDL in macrophages can induce LOX1 expression. Consistent with this, our data indicated that salidroside inhibits THP1-derived foam cell formation by increasing the expression of ABCA1 and decreasing the expression of LOX1. Moreover, Oil Red-O staining showed that salidroside lowered lipid accumulation in THP1 cells in a dose-dependent manner, which is a consequence of the upregulation of ABCA1 and downregulation of LOX1.

With regard to the apoptosis of cells due to oxidative damage, our flow cytometry experiments provided evidence for the apoptosis of THP1 cells in response ox-LDL administration, with a significant increase in the percentages of early and late apoptotic cells. However, upon salidroside treatment, the ox-LDL–induced apoptosis of the cells was reduced in a dose-dependent manner. Hence, it can be considered that the application of salidroside passively reduces the apoptosis caused as a result of oxidative stress.

Oxidative stress can also activate the expression of Nrf2 [42], which is a transcription factor known to upregulate the expression of anti-oxidant enzymes such as HO1, NQO1, and GST [43]. HO1, an anti-oxidative stress factor, is believed to play a cyto-protective role in inflammation and oxidative disorders [44]. For this reason, we tested the effects of salidroside on the endogenous levels of Nrf2 and HO1 expression and found that pretreatment with low concentrations of salidroside (0.1, 1 μM) upregulated Nrf2, but had no effect on HO1 expression. These results suggested that salidroside exerts a cyto-protective effect, possibly by mediating Nrf2 expression.

The MAPK and Akt pathways are mediators of the Nrf2 signaling pathway [45, 46]. Therefore, we detected the activation of the MAPK and Akt in THP1 cells. MAPK is involved in the development of cardiotoxicity and activates downstream signals to further modulate inflammatory responses [47], cell survival, and proliferation [48]. There are three main members in the MAPK family, including ERKs, JNKs, and p38 MAPKs [49, 50]. Several studies have shown that the MAPK pathway is related to the activation of transcription factors including nuclear factor-k-gene binding (NF-κB) and transcription activator-1 (AP-1) [51–53]. These transcription factors regulate the expression of pro-inflammatory genes such as TNF-α, monocyte chemoattractant protein-1 (MCP-1), VCAM-1, and ICAM-1. Interestingly, our former study showed that salidroside increases the expression of ICAM-1 and VCAM-1, which leads to a decrease in neo-intimal or atherosclerotic lesion formation. The Akt pathway is known as an important pro-survival pathway [54], which promotes cell proliferation and angiogenesis and regulates metabolic homeostasis [55], but inhibits apoptosis [56]. Reactive oxygen species (ROS), the by-products of oxidative stress, induce cellular and tissue injury, which in part contribute to the development of several diseases [57, 58]. As an important intracellular second messenger, ROS also dysregulate related signaling pathways to increase stress-induced apoptosis [59]. Activation of the Akt pathway can decrease the levels of ROS, which reverses the progress of atherosclerosis [60].

The extracts of traditional medicinal plants have been found to be effective against anti-oxidative injury, wherein they act by regulating the phosphorylation of the MAPK and Akt pathways [37, 61]. Our findings also shown that pretreatment with salidroside inhibited the ox-LDL–induce increase in the phosphorylation of ERK1/2, JNK, and p38 MAPK in the THP1 cells. Moreover, salidroside also increased the phosphorylated Akt levels in the THP1 cells in a dose-dependent manner.

Recent studies found that MAPK (Mek/ERK1/2) inhibition influences liver X receptor (LXR)-inducible ABCA1 expression in macrophages [62]. In contrast, some other studies have reported that the inhibition of Mek1/2 and Akt could increase the expression of ABCA1 in macrophages [63, 64]. Thus, the effects of the Ras/MAPK pathway on ABC transporter activity have been controversial. Additional studies need to be focused on determining whether salidroside regulates ABCA1 expression in THP1 cells through the MAPK pathway.

## Conclusion

The results from this work show that salidroside can dramatically improve atherosclerosis by inhibiting the formation of foam cells and apoptosis. These beneficial effects are associated with the suppression of oxidative stress, which is brought about by promoting reverse cholesterol transport by upregulating ABCA1 and downregulating LOX1. Our findings also suggest that the MAPK and Akt pathways are involved in the salidroside-induced Nrf2 nuclear translocation, which was indicated by the upregulation of Nrf2 in the THP1 cells.

## Abbreviations

ABCA1: ATP-binding cassette transporter A1
CAD: Coronary artery disease
CO: Carbon monoxide
HO1: Heme oxygenase
LOX1: Lectin-like oxidized low-density lipoprotein receptor-1
MAPK: Mitogen-activated protein kinase
Nrf2: Nuclear factor erythroid 2-related factor 2
ox-LDL: oxidized low-density lipoprotein
PI3ks: Phosphatidylinositide 3-kinases
ROS: Reactive oxygen species

## References

[1] Lindblom R, Ververis K, Tortorella SM, Karagiannis TC (2015). The early life origin theory in the development of cardiovascular disease and type 2 diabetes. Mol Biol Rep.

[2] Tabas I, Williams KJ, Borén J (2007). Subendothelial lipoprotein retention as the initiating process in atherosclerosis: update and therapeutic implications. Circulation.

[3] Seimon TA, Nadolski MJ, Liao X, Magallon J, Nguyen M, Feric NT (2010). Atherogenic lipids and lipoproteins trigger CD36-TLR2-dependent apoptosis in macrophages undergoing endoplasmic reticulum stress. Cell Metab.

[4] Wang Q, Ji J, Hao S, Zhang M, Li K, Qiao T (2016). Iron Together with Lipid Downregulates Protein Levels of Ceruloplasmin in Macrophages Associated with Rapid Foam Cell Formation. Journal of Atherosclerosis. Thrombosis.

[5] Reuland DJ, Mccord JM, Hamilton KL (2013). The role of Nrf2 in the attenuation of cardiovascular disease. Exercise & Sport Sciences Reviews.

[6] Zhu H, Jia Z, Misra BR, Zhang L, Cao Z, Yamamoto M (2008). Nuclear factor E2-related factor 2-dependent myocardiac cytoprotection against oxidative and electrophilic stress. Cardiovasc Toxicol.

[7] Kim KC, Kang KA, Zhang R, Piao MJ, Kim GY, Kang MY (2010). Up-regulation of Nrf2-mediated heme oxygenase-1 expression by eckol, a phlorotannin compound, through activation of Erk and PI3K/Akt. Int J Biochem Cell Biol.

[8] Kaspar JW, Niture SK, Jaiswal AK (2009). Nrf2:INrf2 (Keap1) signaling in oxidative stress. Free Radic Biol Med.

[9] Leonard MO, Kieran NE, Howell K, Burne MJ, Varadarajan R, Dhakshinamoorthy S (2006). Reoxygenation-specific activation of the antioxidant transcription factor Nrf2 mediates cytoprotective gene expression in ischemia-reperfusion injury. FASEB J.

[10] Kensler TW, Wakabayashi N, Biswal S (2007). Cell Survival Responses to Environmental Stresses Via the Keap1-Nrf2-ARE Pathway. Annu Rev Pharmacol Toxicol.

[11] Ding Y, Zhang B, Zhou K, Chen M, Wang M, Jia Y (2014). Dietary ellagic acid improves oxidant-induced endothelial dysfunction and atherosclerosis: role of Nrf2 activation. Int J Cardiol.

[12] Wang WL, Meng ZX, Zhou SJ, Li CJ, Chen R, Lv L (2013). Reduced beta2-glycoprotein I protects macrophages from ox-LDL-induced foam cell formation and cell apoptosis. Lipids Health Dis.

[13] Wang N, Silver DL, Costet P, Tall AR (2000). Specific binding of ApoA-I, enhanced cholesterol efflux, and altered plasma membrane morphology in cells expressing ABC1. J Biol Chem.

[14] Oram JF, Lawn RM, Garvin MR, Wade DP. ABCA1 is the cAMP-inducible apolipoprotein receptor that mediates cholesterol secretion from macrophages. J Biol Chem 2000;275:34508–34511. 10.1074/jbc.M006738200 10918070

[15] Shao B, Tang C, Sinha A, Mayer PS, Davenport GD, Brot N (2014). Humans with atherosclerosis have impaired ABCA1 cholesterol efflux and enhanced high-density lipoprotein oxidation by myeloperoxidase. Circ Res.

[16] Imanishi T, Hano T, Sawamura T, Takarada S, Nishio I (2002). Oxidized low density lipoprotein potentiation of Fas-induced apoptosis through lectin-like oxidized-low density lipoprotein receptor-1 in human umbilical vascular endothelial cells. Circ J.

[17] Sawamura T, Wakabayashi I, Okamura T (2015). LOX-1 in atherosclerotic disease. Clin Chim Acta.

[18] Zhu H, Cao M, Mirandola L, Figueroa JA, Cobos E, Chirivainternati M, Hermonat PL (2014). Comparison of efficacy of the disease-specific LOX1- and constitutive cytomegalovirus-promoters in expressing interleukin 10 through adeno-associated virus 2/8 delivery in atherosclerotic mice. PLoS One.

[19] Nasif WA, Oxidative DNA (2016). damage and oxidized low density lipoprotein in Type II diabetes mellitus among patients with Helicobacter pylori infection. Diabetol Metab Syndr.

[20] Kukongviriyapan U, Apaijit K, Kukongviriyapan V (2016). Oxidative Stress and Cardiovascular Dysfunction Associated with Cadmium Exposure: Beneficial Effects of Curcumin and Tetrahydrocurcumin. Tohoku J Exp Med.

[21] Yazdanparast R, Bahramikia S, Ardestani A (2008). Nasturtium officinale reduces oxidative stress and enhances antioxidant capacity in hypercholesterolaemic rats. Chem Biol Interact.

[22] Hazra B, Biswas S, Mandal N (2008). Antioxidant and free radical scavenging activity of Spondias pinnata. BMC Complement Altern Med.

[23] Yazdanparast R, Ardestani A (2008). vitro antioxidant and free radical scavenging activity of Cyperus rotundus. J Med Food.

[24] Surh YJ, Kundu JK, Na HK, Lee JS (2005). Redox-sensitive transcription factors as prime targets for chemoprevention with anti-inflammatory and antioxidative phytochemicals. J Nutr.

[25] ZQ Q, Zhou Y, Zeng YS, Lin YK, Li Y, Zhong ZQ (2012). Protective effects of a Rhodiola crenulata extract and salidroside on hippocampal neurogenesis against streptozotocin-induced neural injury in the rat. PLoS One.

[26] Huang X, Zou L, Yu X, Chen M, Rui G, Hui C (2015). Salidroside attenuates chronic hypoxia-induced pulmonary hypertension via adenosine A 2a receptor related mitochondria-dependent apoptosis pathway. J Mol Cell Cardiol.

[27] Wei Z, Ming P, Yang Y, Xiao Z, Song B, Lin Z (2015). Protective Effects of Salidroside on Mitochondrial Functions against Exertional Heat Stroke-Induced Organ Damage in the Rat. Evid Based Complement Alternat Med.

[28] Zhang BC, Li WM, Guo R, Xu YW (2012). Salidroside decreases atherosclerotic plaque formation in low-density lipoprotein receptor-deficient mice. Evid Based Complement Alternat Med.

[29] Mishra BB, Tiwari VK (2011). Natural products: an evolving role in future drug discovery. Eur J Med Chem.

[30] Paterson I, Anderson EA (2005). Chemistry. The renaissance of natural products as drug candidates. Science.

[31] Kucinskaite A, Briedis V, Savickas A (2004). Eksperimentiniai augalo Rhodiola rosea L. Medicina (Kaunas).

[32] Daigneault M, Preston JA, Marriott HM, Whyte MK, Dockrell DH (2010). The Identification of Markers of Macrophage Differentiation in PMA-Stimulated THP-1 Cells and Monocyte-Derived Macrophages. PLoS One.

[33] Chen KS, Chen PN, Hsieh YS, Lin CY, Lee YH, Chu SC (2015). Capsaicin protects endothelial cells and macrophage against oxidized low-density lipoprotein-induced injury by direct antioxidant action. Chem Biol Interact.

[34] Curtin JF, Donovan M, Cotter TG (2002). Regulation and measurement of oxidative stress in apoptosis. J Immunol Methods.

[35] Mukherjee N, Parida PK, Santra A, Ghosh T, Dutta A, Jana K (2016). Oxidative stress plays major role in mediating apoptosis in filarial nematode Setaria cervi in the presence of trans-stilbene derivatives. Free Radic Biol Med.

[36] Seimon T, Tabas I (2009). Mechanisms and consequences of macrophage apoptosis in atherosclerosis. J Lipid Res.

[37] Zhu M, Li J, Wang K, Hao X, Ge R, Li Q (2016). Isoquercitrin Inhibits Hydrogen Peroxide-Induced Apoptosis of EA.hy926 Cells via the PI3K/Akt/GSK3β Signaling Pathway. Molecules.

[38] Thorp E, Li G, Seimon TA, Kuriakose G, Ron D, Tabas I (2009). Reduced Apoptosis and Plaque Necrosis in Advanced Atherosclerotic Lesions of Apoe −/− and Ldlr −/− Mice Lacking CHOP. Cell Metab.

[39] Wu M, Liu M, Guo G, Zhang W, Liu L (2015). Polydatin Inhibits Formation of Macrophage-Derived Foam Cells. Evid Based Complement Alternat Med.

[40] Cho W, Kang JL, Park YM (2015). Corticotropin-Releasing Hormone (CRH) Promotes Macrophage Foam Cell Formation via Reduced Expression of ATP Binding Cassette Transporter-1 (ABCA1). PLoS One.

[41] Thakkar S, Wang X, Khaidakov M, Dai Y, Gokulan K, Mehta JL (2015). Structure-based Design Targeted at LOX-1, a Receptor for Oxidized Low-Density Lipoprotein. Sci Rep.

[42] Esakky P, Hansen DA, Drury AM, Moley KH (2015). Cigarette smoke-induced cell cycle arrest in spermatocytes [GC-2spd(ts)] is mediated through crosstalk between Ahr–Nrf2 pathway and MAPK signaling. J Mol Cell Biol.

[43] Park SY (2011). Park dJ, Kim YH, Kim Y, Kim SG, Shon KJ, et al. Upregulation of heme oxygenase-1 via PI3K/Akt and Nrf-2 signaling pathways mediates the anti-inflammatory activity of Schisandrin in Porphyromonas gingivalis LPS-stimulated macrophages. Immunol Lett.

[44] Bobermin LD, Wartchow KM, Flores MP, Leite MC, Quincozes-Santos A, Gonçalves CA (2015). Ammonia-induced oxidative damage in neurons is prevented by resveratrol and lipoic acid with participation of heme oxygenase 1. Neurotoxicology.

[45] Sun Z, Huang Z, Zhang DD (2009). Phosphorylation of Nrf2 at multiple sites by MAP kinases has a limited contribution in modulating the Nrf2-dependent antioxidant response. PLoS One.

[46] Shi X, Zhou B (2010). The role of Nrf2 and MAPK pathways in PFOS-induced oxidative stress in zebrafish embryos. Toxicol Sci.

[47] Bi Q, Hou J, Qi P, Ma C, Feng R, Yan B (2016). TXNIP/TRX/NF-κB and MAPK/NF-κB pathways involved in the cardiotoxicity induced by Venenum Bufonis in rats. Sci Rep.

[48] Du W, Pang C, Xue Y, Zhang Q, Wei X (2015). Dihydroartemisinin inhibits the Raf/ERK/MEK and PI3K/AKT pathways in glioma cells. Oncol Lett.

[49] Roux PP, Blenis J. ERK and p38 MAPK-activated protein kinases: a family of protein kinases with diverse biological functions. Microbiol Mol Biol Rev 2004; 68:320–344. 10.1128/MMBR.68.2.320-344.2004 PMC419926 15187187

[50] Mebratu Y, Tesfaigzi Y (2009). How ERK1/2 Activation Controls Cell Proliferation and Cell Death Is Subcellular Localization the Answer?. Cell Cycle.

[51] Rawadi G, Garcia J, Lemercier B, Romanroman S (1999). Signal transduction pathways involved in the activation of NF-kappa B, AP-1, and c-fos by Mycoplasma fermentans membrane lipoproteins in macrophages. J Immunol.

[52] Li LB, Leung DY, Goleva E (2015). Activated p38 MAPK in Peripheral Blood Monocytes of Steroid Resistant Asthmatics. PLoS One.

[53] Oeckinghaus A, Hayden MS, Ghosh S (2011). Crosstalk in NF-κB signaling pathways. Nat Immunol.

[54] Jie P, Hong Z, Tian Y, Li Y, Lin L, Zhou L (2015). Activation of transient receptor potential vanilloid 4 induces apoptosis in hippocampus through downregulating PI3K|[sol]|Akt and upregulating p38 MAPK signaling pathways. Cell Death Dis.

[55] Matthews AT, Ross MK (2015). Oxyradical Stress, Endocannabinoids, and Atherosclerosis. Toxics.

[56] Zhang Y, Huang S, Leng Y, Chen X, Liu T, Wang H (2016). Effect of DcR3-specific siRNA on cell growth suppression and apoptosis induction in glioma cells via affecting ERK and AKT. Onco Targets Ther.

[57] Kataoka Y, Puri R, Nicholls SJ (2015). Inflammation, plaque progression and vulnerability: evidence from intravascular ultrasound imaging. Cardiovasc Diagn Ther.

[58] Van dLB, Labugger R, Skepper JN, Bachschmid M, Kilo J, Powell JM, et al. Enhanced Peroxynitrite Formation Is Associated with Vascular Aging. J Exp Med 2011;192(12):1731–1744. 10.1084/jem.192.12.1731 PMC2213492 11120770

[59] Elmas MM, Fan M, Abdelrahman AA (2013). Role of rostral ventrolateral medullary ERK/JNK/p38 MAPK signaling in the pressor effects of ethanol and its oxidative product acetaldehyde. Alcohol Clin Exp Res.

[60] Beach A, Richard VR, Bourque S, Boukhviner T, Kyryakov P, Gomezperez A, Arliaciommo A, Feldman R, Leonov A, Piano A (2015). Lithocholic bile acid accumulated in yeast mitochondria orchestrates a development of an anti-aging cellular pattern by causing age-related changes in cellular proteome. Cell Cycle.

[61] Kaewthawee N, Brimson S (2013). The effects of ursolic acid on cytokine production via the MAPK pathways in leukemic T-cells. EXCLI J.

[62] Mulay V, Wood P, Manetsch M, Darabi M, Cairns R, Hoque M (2013). Inhibition of mitogen-activated protein kinase Erk1/2 promotes protein degradation of ATP binding cassette transporters A1 and G1 in CHO and HuH7 cells. PLoS One.

[63] Dong F, Mo Z, Eid W, Courtney KC, Zha X (2013). Akt Inhibition Promotes ABCA1-Mediated Cholesterol Efflux to ApoA-I through Suppressing mTORC1. PLoS One.

[64] Zhou X, Yin Z (2010). Inhibition of ERK1/2 and activation of liver X receptor synergistically induce macrophage ABCA1 expression and cholesterol efflux. J Biol Chem.

